# SetD7 (Set7/9) is a novel target of PPARγ that promotes the adaptive pancreatic β-cell glycemic response

**DOI:** 10.1016/j.jbc.2021.101250

**Published:** 2021-09-28

**Authors:** Thomas L. Jetton, Patricio Flores-Bringas, John L. Leahy, Dhananjay Gupta

**Affiliations:** Division of Endocrinology, Diabetes and Metabolism, Department of Medicine, Larner College of Medicine, University of Vermont, Burlington, Vermont, USA

**Keywords:** type 2 diabetes (T2D), SetD7 (Set7/9), PPARγ, β-cell compensation, thiazolidinedione (TZD), gene transcription, partial pancreatectomy (Px);, high-fat diet, obesity, β-cell mass, glucose homeostasis, ChIP, chromatin immunoprecipitation assay, EMSA, electrophoretic mobility shift assay, HFD, high-fat diet, H3K4, histone-3-lysine-4, KD, knockdown, PPARγ, peroxisome proliferator-activated receptor-gamma, PPRE, PPARγ response element, SET, Su (var), Enhancer of zeste, Trithorax, TF, transcription factor, T2D, type 2 diabetes

## Abstract

Loss of functional pancreatic β-cell mass leads to type 2 diabetes (T2D), attributable to modified β-cell-dependent adaptive gene expression patterns. SetD7 is a histone methyltransferase enriched in pancreatic islets that mono- and dimethylates histone-3-lysine-4 (H3K4), promoting euchromatin modifications, and also maintains the regulation of key β-cell function and survival genes. However, the transcriptional regulation of this important epigenetic modifier is unresolved. Here we identified the nuclear hormone receptor peroxisome proliferator-activated receptor-gamma (PPARγ) as a major transcriptional regulator of *SetD7* and provide evidence for direct binding and functionality of PPARγ in the *SetD7* promoter region. Furthermore, constitutive shRNA-mediated *PPARγ* knockdown in INS-1 β-cells or pancreas-specific *PPARγ* deletion in mice led to downregulation of SetD7 expression as well as its nuclear enrichment. The relevance of the SetD7-PPARγ interaction in β-cell adaptation was tested in normoglycemic 60% partial pancreatectomy (Px) and hyperglycemic 90% Px rat models. Whereas a synergistic increase in islet PPARγ and SetD7 expression was observed upon glycemic adaptation post-60% Px, in hyperglycemic 90% Px rats, islet *PPARγ*, and PPARγ targets *SetD7* and *Pdx1* were downregulated. PPARγ agonist pioglitazone treatment in 90% Px rats partially restored glucose homeostasis and β-cell mass and enhanced expression of *SetD7* and *Pdx1*. Collectively, these data provide evidence that the SetD7-PPARγ interaction serves as an important element of the adaptive β-cell response.

Type 2 diabetes mellitus (T2D) is a progressive metabolic disorder in which defective β-cell adaptive responses are coupled with insulin resistance and genetic susceptibility to be the main pathological contributors (1, 2, 3, 4, 5). Pancreatic β-cells are key mediators of glucose homeostasis and are endowed with comprehensive adaptive mechanisms for augmenting their mass and function in order to meet the physiological demands for insulin (4, 5). Identification of pathways for successful *versus* failed β-cell adaptation is critical to design innovative interventions for diabetes prevention (5). Transcription factors (TFs) play a central role in the adaptive changes in β-cell mass and function by acting as regulatory switches for key gene expression (6, 7). A precise understanding of how the metabolic environment is linked to the flow of genetic information through opened and closed conformations of chromatin during the β-cell adaptive response is still not clear. Recent studies have demonstrated considerable developmental plasticity in islet cell types, and supportive data suggest that β-cell dedifferentiation is a pathogenic feature of T2D (8, 9, 10, 11). Thus, a more thorough understanding of epigenetic markers that define β-cell identity and functional preservation may provide important insight into novel approaches for precise manipulation of cellular programming to restore loss of β-cell identity (12).

SetD7 (Set7/9) is a member of the SET (Su (var), Enhancer of zeste, Trithorax) family of histone methyltransferases. The crystal structure and biochemical analyses have shown that SetD7 mono- and dimethylates histone3 lysine 4 (H3K4) and promotes the transcriptionally favorable open chromatin conformation1 (13, 14, 15). SetD7 expression is mainly restricted to pancreatic islets (β-cells, α-cells, but occasionally in some duct cells) in the mature murine and human pancreas (16, 17). Depletion of SetD7 expression by siRNA in islets and insulinoma cells is reported to cause repression of *Pdx1* regulated *Glut2*, *Ins1/Ins2*, and *MafA* gene expression involved with glucose sensing and glucose-stimulated insulin secretion (17). Additionally, the transcriptional downregulation of *Ins1/Ins2* and *Glut2* genes has been associated with loss of dimethylated H3Lys4 and RNA polymerase recruitment in the promoter regions of *Ins1/Ins2* and *Glut2* (17, 18). A mouse model with MIP1-Cre^ERT^-driven β-cell-specific knockout of SetD7 developed glucose intolerance with islet specific downregulation of the critical β-cell genes *Pdx1*, *MafA*, *Glut2*, *and Gck* (18). Combined together, these studies have demonstrated that SetD7-mediated regulation of β-cell specific gene expression is required for their normal function and survival. However, importantly, there is a lack of information on the upstream regulation of SetD7 and its role in the functional adaptation of β-cell mass and function.

We previously mapped the β-cell physiological adaptation responses that allow maintenance of glucose homeostasis in a normoglycemic rodent model of 60% partial pancreatectomy (60%-Px) (19, 20, 21, 22, 23). We identified the sequential activation of a network of TFs with FoxO1 and PPARγ acting as crucial initiators of the β-cell compensation response to regulate expression of β-cell effector genes (24). The identified downstream gene targets of PPARγ include *Pdx* (25) (β-cell differentiation, function, and survival), *Gipr* (26) (incretin-regulated insulin secretion), *pyruvate carboxylase* (27) (mitochondrial fuel metabolism), *Glut2* (28) and *Gck* (29) (glucose sensing), *Gpr40* (30, 31) (fatty acid induced insulin secretion), *Serca2A* (32) (β-cell calcium homeostasis), and FGF21 action (33). Previously, Evans-Molina *et al.* (34) demonstrated that treatment of mouse models of diabetes and obesity with the PPARγ agonist pioglitazone improved glycemia with enhanced expression of Pdx1 and SetD7 proteins. However, a mechanistic explanation of heightened SetD7 expression and its putative role in adaptive β-cell response is not resolved.

In this study, we provide comprehensive *in vitro* and *in vivo* evidence for PPARγ-mediated direct transcriptional regulation of *SetD7* expression in β-cells. We also show that the molecular interaction between PPARγ and SetD7 plays a key role in the adaptive β-cell response in the background of experimental loss of β-cell mass in rats. We have measured temporal changes in *SetD7* expression in a mouse model undergoing high-fat diet (HFD)-induced chronic metabolic stress. Additionally, we characterized the effects of constitutive knockdown (KD) of *SetD7* on key β-cell prodifferentiation and profunction maker Pdx1, glucose-stimulated insulin secretion, and β-cell proliferation.

## Results

### PPARγ occupies the SetD7 promoter

**Figure 1:**
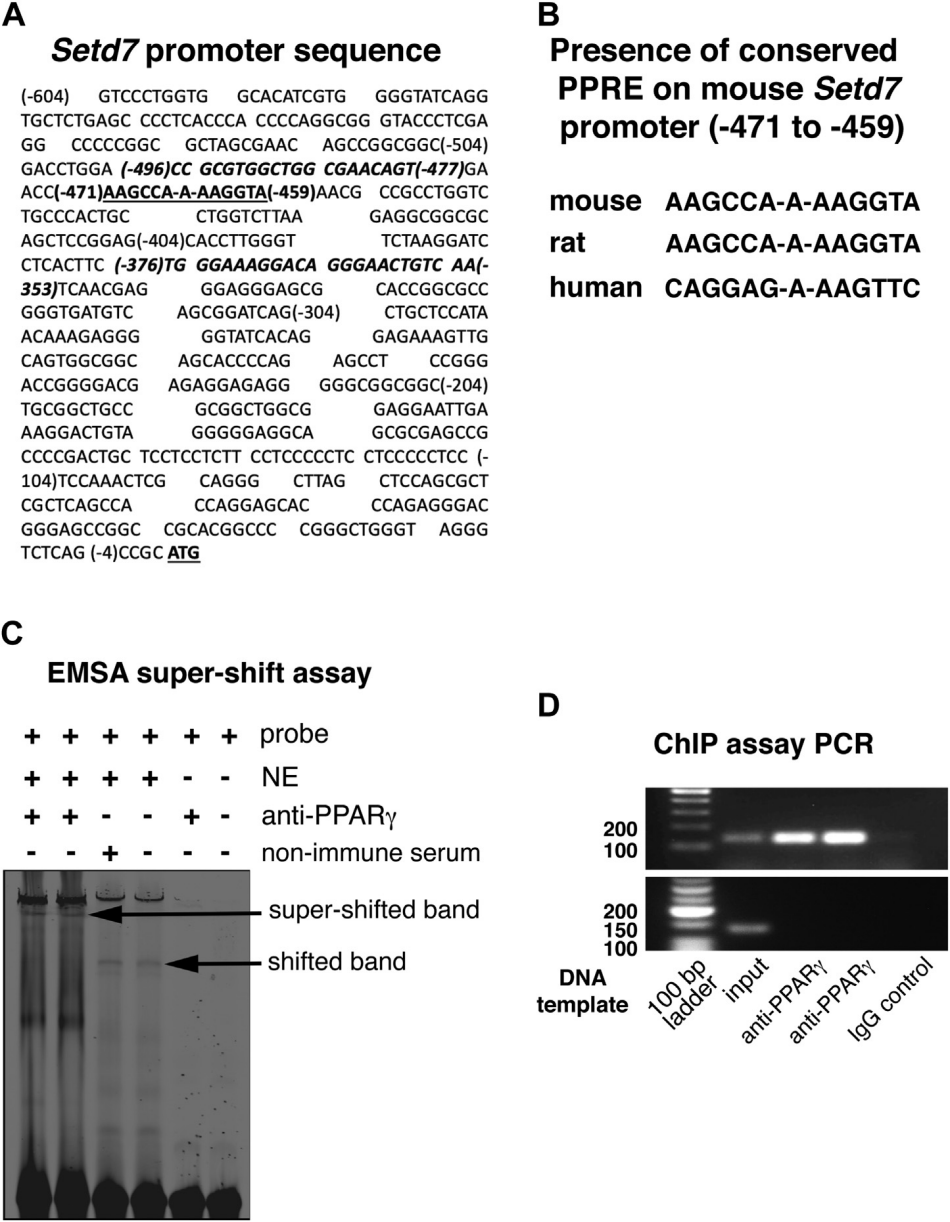
**Nuclear binding assays.***A*, a partial *SetD7* mouse promoter sequence curated using NM_080,793 as DNA reference sequence. The *SetD7* PPRE sequence is *bolded* and *underlined*. The DNA sequences used for the primers designed for the ChIP assay PCR are *bolded* and *italicized*. *B*, consensus PPRE sequence on the *SetD7* promoter: A putative PPARγ response element was found using MatInspector software on the *SetD7* promoter region that was conserved between mouse, rat, and human SetD7 promoter sequences. *C*, EMSA and super-shift assay of *SetD7* PPRE: PAGE purified infrared dye (IRD-700) tagged oligos for *SetD7* PPRE were annealed and EMSA was performed using nuclear extracts prepared from INS-1 cells as detailed in the Experimental procedures section. For the super-shift assay, INS-1 cell nuclear extracts were preincubated with a PPARγ specific antibody or mouse nonimmune serum on ice for 30 min before adding infrared dye (IRD-700) tagged oligos for *SetD7* PPRE. *D*, *upper panel*, ChIP assay PCR showing 144 base pairs (bp) *SetD7* PPRE positive band: 300–400 bp chromatin preparations of βTC6 cells were prepared as described in the Experimental procedures section. The prepared chromatin samples were immunoprecipitated using anti-PPARγ *versus* a control nonimmune IgG, followed by PCR of the immunoprecipitated and nonimmunoprecipitated DNA (input DNA) using flanking primer pairs to the mouse SetD7-PPRE. The shown representative bands are the 144 bp (expected length) PCR product from two separate chromatin preparations (out of three independent analysis), along with absence of PCR product in the control mouse IgG lane. *D*, *lower panel*, ChIP assay PCR run with input DNA and digested chromatin precipitated with PPARγ specific antibody using primer pairs 800 bp from the *SetD7* PPRE with expected PCR product of 129 bp length. The data presented showing the PCR product with input DNA, but the absence in digested chromatin precipitated with PPARγ specific antibody or mouse IgG, thus confirming the fidelity of the ChIP assay PCR in terms of target specificity.

The PPARγ agonist pioglitazone increases the expression of SetD7 in β-cells (34). To investigate the possibility of direct transcriptional regulation of *SetD7* by PPARγ, we curated the *SetD7* promoter sequence using NM_080,793 as DNA reference sequence. The *SetD7* promoter sequence was validated with Eukaryotic Promoter Database (EPD). A segment of *SetD7* promoter sequence is presented in Figure 1*A*. The curated *SetD7* promoter sequence was analyzed by Genomatix MatInspector software and identified conserved putative PPARγ response elements (PPREs) in the mouse (underlined in Fig. 1*A*), rat and human promoter sequences (Fig. 1*B*). We next performed electrophoretic mobility shift assays (EMSA) with INS1 β-cell nuclear extracts (NE) and the annealed mouse dsDNA *SetD7* PPRE sequence. The *SetD7* PPRE with NE showed a shifted band, and we confirmed PPARγ-specific binding by addition of an anti-PPARγ antibody that resulted in a super-shifted complex (Fig. 1*C*). The *in vivo* binding of PPARγ on the *SetD7* promoter was confirmed by performing chromatin immunoprecipitation assay (ChIP) in mouse-derived βTC6 cells treated with 10 μM pioglitazone for 48 h. The mouse-derived βTC6 cells were used for the ChIP assay since the ChIP assay primers were designed covering the mouse *SetD7* PPRE using the mouse *SetD7* promoter sequences (Fig. 1*A*, the primer pairs are presented in italicized font) Using a PPARγ specific antibody, we pulled down a PPARγ-DNA complex. The ChIP PCR using primer pairs covering the putative *SetD7* PPRE region generated a predicted 144 bp DNA PCR product confirming a PPARγ specific complex on the *SetD7* promoter (Fig. 1*D*, upper panel). These findings demonstrate promoter occupancy of PPARγ on the *SetD7* gene. The chromatin immunoprecipitated by using PPARγ specific antibody and IgG was further probed with ChIP PCR using primer pair covering *Setd7* promoter region 800 base pairs from the *Setd7* PPRE with the expected ChIP PCR product of 129 base pairs. The absence of ChIP PCR using non-PPRE specific primers validates the fidelity of PPARγ-specific chromatin enrichment on the designated PPRE on the *Setd7* promoter (Fig. 1*D*, lower panel).

### SetD7 expression is downregulated in the context of PPARγ inactivity

**Figure 2:**
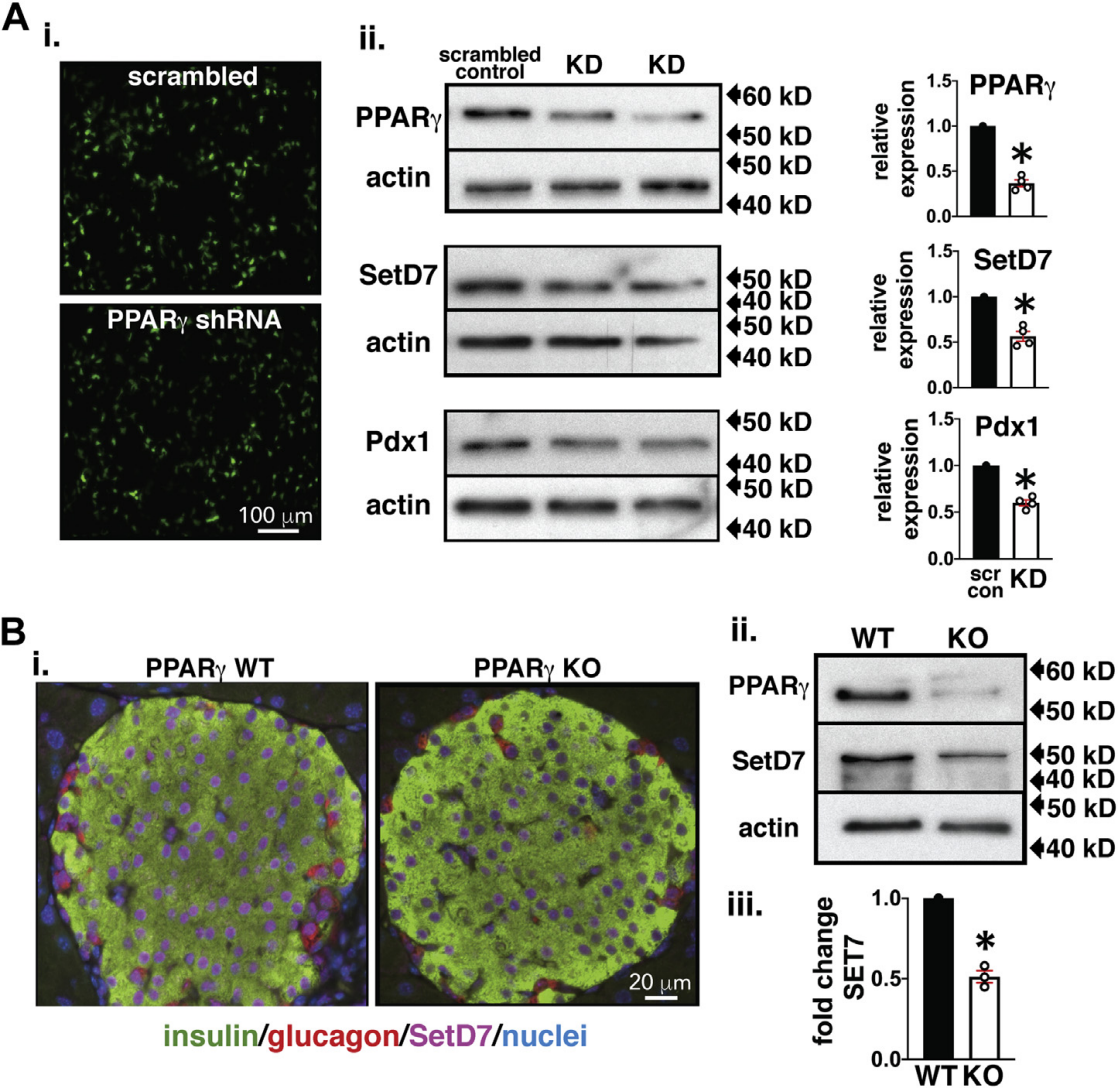
**SetD7 is reduced in PPARγ KD and PANC PPARγ KO β-cells.***A*, *i*, 10× overview of live INS-1 cells expressing GFP-tagged PPARγ shRNA or scrambled (Scr) shRNA. *A*, *ii*, representative WB and band quantitation for PPARγ, SetD7 and Pdx1 relative to the scrambled control, ∗*p* < 0.001. *B*, *i*, Representative immunofluorescence fields, imaged were under identical conditions, from 8-week-old WT and PANC PPARγ KO islets stained for insulin (*green*), SetD7 (*magenta*), and glucagon (*red*). *B*, *ii*, representative WB showing protein levels of PPARγ and SetD7 in the islets of 8-week-old WT and PANC PPARγ KO mice. *B*, *iii*, quantitative band analysis of SetD7 in WT and PANC PPARγ KO mice. ∗*p* < 0.001.

Previously, we reported the presence of a functional PPRE within the *Pdx1* promoter (25) and thus used Pdx1 expression as a parallel downstream marker while evaluating SetD7 transcriptional regulation by PPARγ. We next tested direct regulation of SetD7 by PPARγ in INS-1 cells with a constitutively KD of *PPARγ*. We established and characterized GFP-tagged stable constitutive expression of *PPARγ* shRNA (Origene) and scrambled control (scr) in INS-1 β-cells (Fig. 2*Ai*). We obtained an shRNA clone that reduced PPARγ protein expression to 0.37-fold and correspondingly reduced Pdx1 (0.6-fold) and SetD7 (0.57-fold) relative to the scr control (Fig. 2*Aii*). These data demonstrate that SetD7 is downstream of PPARγ signaling. Previously, we characterized pancreas-specific *PPARγ* KO mice that exhibited hyperglycemia and impaired glucose-induced insulin secretion (25). We also observed downregulation of *Pdx1* expression basally and an attenuated thiazolidinedione (TZD)-mediated transcriptional upregulation of *Pdx1* gene expression in the islets of PPARγ KO mice compared with wild-type littermate islets (25). Here we evaluated the impact of islet-specific KO of *PPARγ* on SetD7 expression. Immunofluorescence imaging of pancreatic sections from PANC-*PPARγ* KO mice clearly demonstrated reduced nuclear SetD7 signal compared with the wild-type littermates (Fig. 2*Bi*). Additionally, WB data of PANC-*PPARγ* islets showed downregulation of SetD7 protein expression (0.5-fold) (Fig. 2*B*, *ii* and *iii*). Collectively, these *in vitro* and *in vivo* data strongly support that PPARγ is an upstream regulator of SetD7.

### PPARγ agonist stimulates SetD7 expression

**Figure 3:**
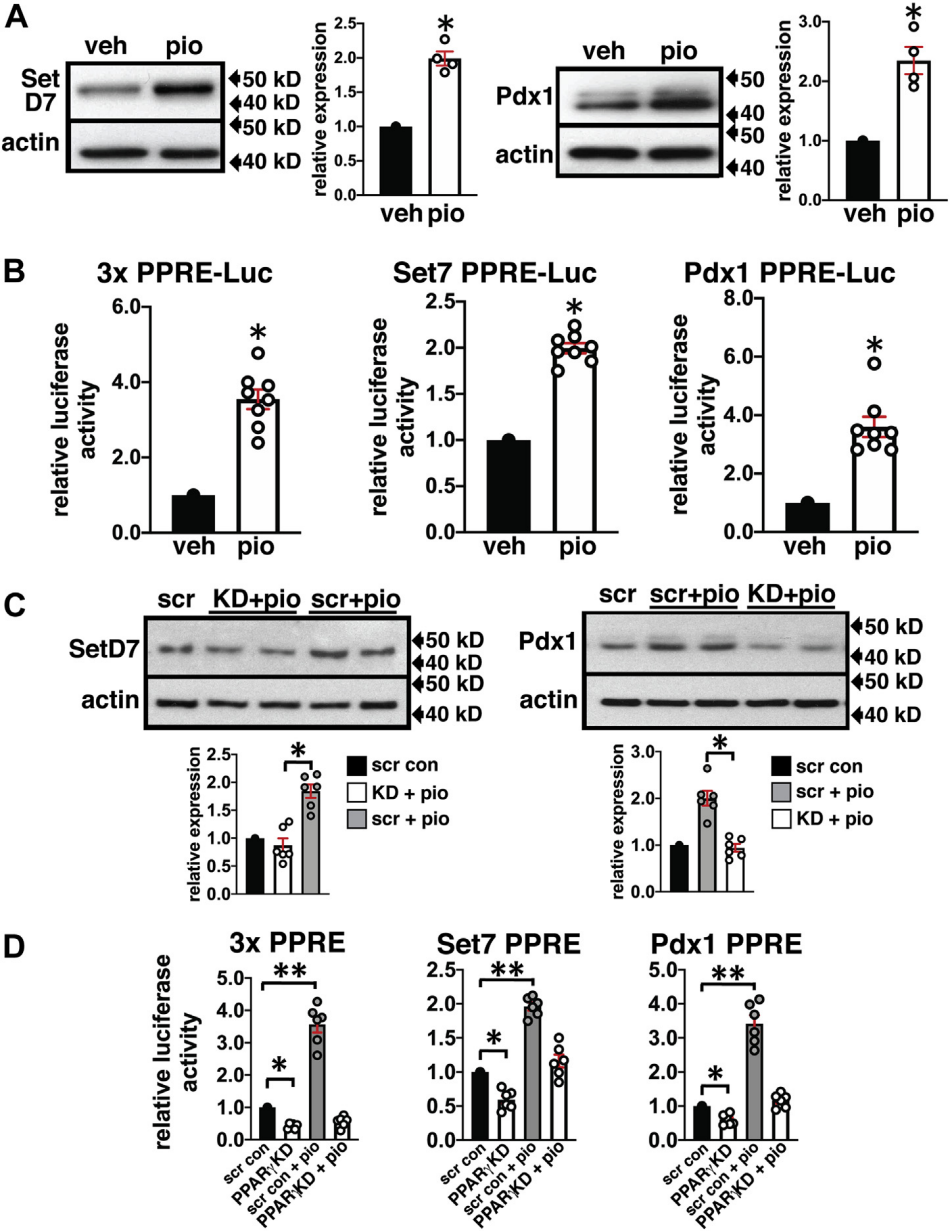
**Pioglitazone-mediated activation of PPARγ upregulates protein expression and promoter reporter activities of SetD7 and Pdx1 that is attenuated in *PPARγ* KD INS-1 cells.***A*, representative WB showing increased expression of SetD7 and Pdx1 by the PPARγ agonist-pioglitazone compared with vehicle treated INS-1 cells: INS-1 cells were treated with 10 μM pioglitazone or DMSO for 72 h. Cell lysates were prepared and processed for WB analysis for SetD7 and Pdx1 with β-actin used for protein loading normalization. Band quantitation was performed using NIH-ImageJ, and the data presented is representative of four separate blots of SetD7 and Pdx1. ∗*p* < 0.01. *B*, promoter reporter assays for PPARγ, SetD7, and Pdx1: INS-1 cells transfected with 3xPPRE-Luc, SteD7-PPRE-Luc, and Pdx1-PPRE-Luc promoter reporter vectors. The *Renilla* luciferase reporter plasmid was included in all transfections serving as an internal control. Twenty-four hours post transfection, cells were treated with 10 μM pioglitazone or DMSO for 48 h. Firefly luciferase activity was measured by a luminometer, normalized with *Renilla* luciferase, and expressed as relative luciferase activity compared with the vehicle-treated experimental group. The data presented are representative of three independent experiments. ∗*p* < 0.00001. *C*, representative WB showing induction of SetD7 and Pdx1 protein levels in the scr control INS-1 cells treated with 10 μM pioglitazone for 72 h relative to vehicle (DMSO) control; however, INS-1 cells with PPARγ KD lacked pioglitazone responsiveness to modulate SetD7 and Pdx1 protein levels. The WB quantitation data is representative of n = 6 samples. ∗*p* < 0.001. *D*, promoter reporter assays for PPARγ, SetD7, and Pdx1 in *PPARγ* shRNA INS-1 cells compared with Scr shRNA expressing INS-1 cells treated with vehicle or pioglitazone. ∗*p* < 0.05, ∗∗*p* < 0.01.

We next evaluated the functional implications of the transcriptional activation of *SetD7* by the PPARγ agonist. INS-1 cells were incubated 72 h with vehicle (DMSO) or 10 μM pioglitazone. WB analysis of cell lysates showed 2-fold increases in SetD7 and Pdx1 protein expression (Fig. 3*A*). Next, INS-1 cells were transfected with *PPARγ*, *SetD7*, and *Pdx1* promoter luciferase constructs carrying a *PPARγ* PPRE. Transfected cells were treated with DMSO or 10 μM pioglitazone for 48 h. We performed dual reporter assays (Promega kit) at 48 h posttransfection and normalized the PPRE-mediated luciferase activity with *Renilla* activity. Compared with the vehicle control, the pioglitazone-treated β-cells exhibited 3.5-fold, 2-fold, and 3-fold increases in the respective reporter activities of *PPARγ*, *SetD7*, and *Pdx1* (Fig. 3*B*). These data provide evidence for pioglitazone-mediated activation of PPARγ leading to enhance promoter reporter activities of *Pdx1* and *SetD7* with a similar fold upregulation of Pdx1 and SetD7 protein expression. We next measured the protein expression of SetD7 and Pdx1 in INS-1 cells with scr and *PPARγ* KD treated with vehicle (DMSO) or 10 μM pioglitazone for 72 h. We observed a 2-fold upregulations of SetD7 and Pdx1 protein levels in the scr INS-1 cells treated with pioglitazone, whereas INS-1 cells with *PPARγ* KD lost the stimulatory effects of pioglitazone (Fig. 3*C*). We measured *PPAR*γ, *SetD7*, and *Pdx1* promoter reporter activities under similar experimental conditions described above (Fig. 3*D*) and observed downregulation of basal levels of *PPARγ* (0.4-fold), *SetD7* (0.6-fold), and *Pdx1* (0.64-fold) reporter activities in the *PPARγ* KD INS-1 β-cells compared with scr-INS-1 cells. Importantly, pioglitazone-mediated stimulation of *PPARγ*, *SetD7*, and *Pdx1* promoter reporter activities was also attenuated with KD of *PPARγ* (Fig. 3*D*). These data provide evidence for the dependency of *Pdx1* and *SetD7* promoter activities and subsequent protein expression on transcriptional activation of *PPARγ* by its agonist pioglitazone.

### Constitutive KD of SetD7 in INS-1 cells downregulates Pdx1, reduces insulin secretion, and increases proliferation

**Figure 4:**
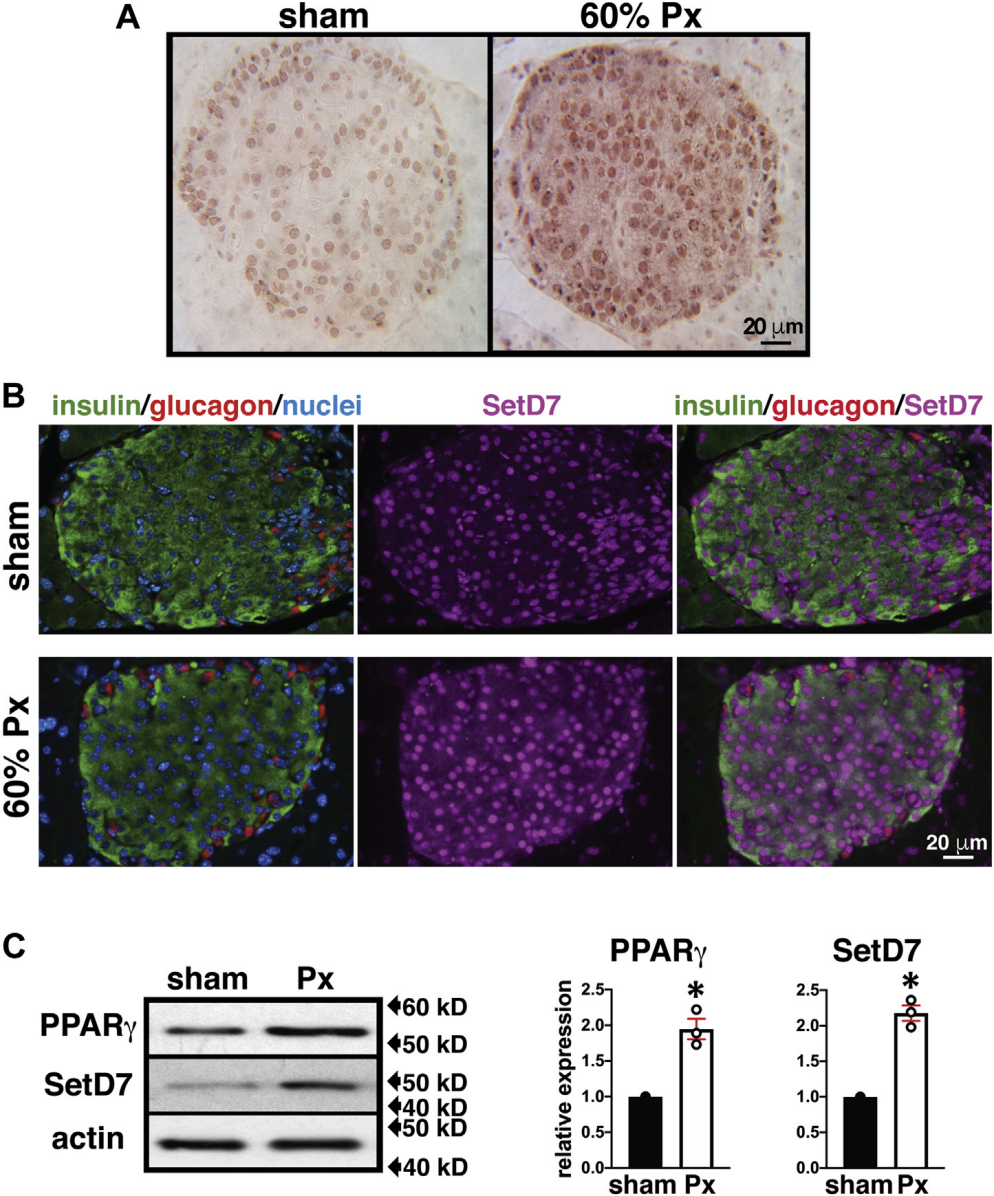
**Characterization of constitutive KD of *SetD7* in INS-1 cells and temporal measurements of *SetD7* expression in a mouse model of HFD-induced metabolic stress.***A*, low power overview of live INS-1 cells expressing RFP-tagged *SetD7* shRNA or scrambled (Scr) shRNA showing RFP positive expression of scrambled and shRNA against *SetD7* expression. *B*, representative WB and band quantitation for SetD7 and Pdx1 in *SetD7* KD INS-1 cells relative to the scrambled control, ∗*p* < 0.01. *C*, measurement of the glucose-stimulated insulin secretion (GSIS) in the scr control *versus SetD7* KD INS-1 cells. The INS-1 cells were primed for GSIS following 12 h incubation in the complete RPMI media with 2.8 mM glucose followed by equilibration for 2 h in warm and oxygenated KRBH pH 7.4. The GSIS was performed with low glucose (2.8 mM) and high glucose (16.7 mM) for 1 h in KRBH pH 7.4. Secreted insulin was measured by ELISA. The secreted insulin data was normalized with total protein content in each well. The scr shRNA control INS-1 cells under high glucose (16.7 mM) exhibited an ∼4.8-fold increase in GSIS (∗*p* < 0.002), whereas *SetD7* KD INS-1 cells demonstrated a modest ∼2.1-fold increase in GSIS (∗∗*p* < 0.001). *D*, proliferation frequency assay in scr control *versus SetD7* KD INS-1 cells measured by BrdU incorporation, ∗*p* < 0.0001. *E*, *SetD7* mRNA levels were evaluated (1–16 weeks) in isolated islets of mice undergoing control diet or high-fat feeding. Total RNA preparations were processed for RT-PCR for the expression of *SetD7* using FAM-tagged rat PCR primers. The CT values were normalized with β-actin. The *SetD7* mRNA expression data presented is relative to age-matched control chow-fed mice and demonstrates a peak in islet *SetD7* at 8 weeks HFD corresponding with the height of β-cell functional compensation followed by a marked reduction by 16 weeks corresponding with β-cell decompensation (35). ^#^*p* < 0.0001 compared with all other time points; ^##^*p* < 0.05 compared with weeks 3–8.

We have provided evidence for upstream transcriptional regulation of *Pdx1* (25) and *SetD7* by PPARγ (Figure 1, Figure 2, Figure 3). However, previous reports have also demonstrated that *SetD7* KD in islets downregulate *Pdx1* (18). We established constitutive KD of *SetD7* in INS-1 cells to further examine the role of SetD7 in the regulation of *Pdx1* as a key β-cell differentiation, function, and survival marker. We obtained constitutive KD of *SetD7* INS-1 cells using an RFP-tagged shRNA plasmid against *SetD7* (Fig. 4*A*) showing a very effective 66% downregulation of SetD7 compared with scr shRNA control INS-1 cells (Fig. 4*B*). The constitutive KD of *SetD7* also downregulated Pdx1 (0.49-fold) compared with scr shRNA control INS-1 cells. We next measured the functional impact of *SetD7* KD by performing glucose-stimulated insulin secretion (GSIS) analysis. The scr shRNA control INS-1 cells under high glucose (16.7 mM) exhibited an ∼4.8-fold increase in GSIS (*p* < 0.002), whereas *SetD7* KD INS-1 cells demonstrated a modest ∼2.1-fold increase in GSIS (*p* < 0.001) (Fig. 4*C*). Next, we measured the effect of *SetD7* KD on INS-1 cell proliferation by a BrdU incorporation assay using a standard 6 h "pulse". Compared with scr shRNA control INS-1 cells, the *SetD7* KD increased INS-1 cell proliferation approximately 2-fold (*p* < 0.0001) (Fig. 4*D*). Combined together, these supportive data suggest that *SetD7* KD in INS-1 cells directly affects β-cell differentiation and Pdx1 expression causing a shift from profunction and differentiation to increased proliferation.

### SetD7 expression under metabolic stress of a high-fat diet

Next, we evaluated *SetD7* expression profile in the islets of HFD-induced metabolic stress in mice described previously (35). The *SetD7* mRNA levels were measured in the isolated islets in a temporal manner from 1 to 16 weeks of HFD and control (Fig. 4*E*). *SetD7* mRNA levels were downregulated within a week of HFD that coincided with development of early glucose intolerance in the HFD mice (35). However, by second to fourth week, *SetD7* mRNA was restored to control levels preceded by increase in *PPARγ* mRNA (35). During the peak of the metabolic β-cell compensation under HFD (8 week), *SetD7* mRNA reached maximal levels of 1.7-fold expression relative to chow-fed controls that coincided with previously reported increase in mRNA levels of *PPARγ* and *Pdx1* as observed previously (35). However, under persistent metabolic stress, the decompensatory phase of β-cell response after 12 weeks of HFD was marked with downregulation of *SetD7* mRNA compared with control diet-fed mice (Fig. 4*E*). The temporal changes in *SetD7* expression under HFD demonstrate that *SetD7* is an integral component of the β-cell compensatory response with a synergistic increase in *PPARγ expression* as observed previously (35).

### *In vivo* evidence for parallel changes in PPARγ and SetD7 expression and nuclear enrichment in β-cells from rats undergoing successful or failed adaptive responses to the abrupt loss of β-cell mass

**Figure 5:**
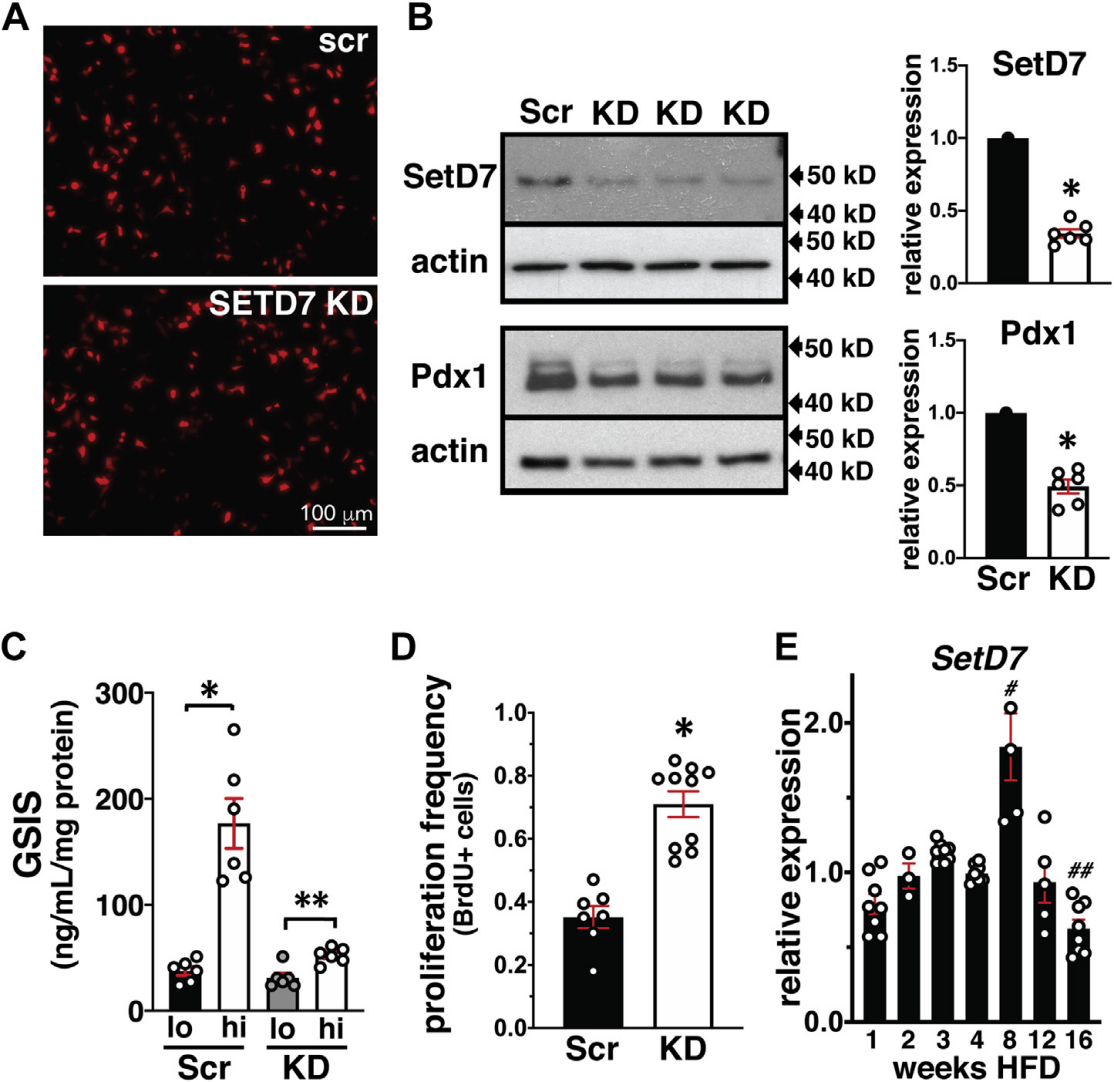
**SetD7 expression in β-cells of sham and 60% Px in rats post 14-days surgery.***A*, SetD7 immunohistochemistry: Pancreatic sections of sham and 60% Px rats were stained for SetD7 (indirect immunoperoxidase staining). Representative pancreatic islet fields demonstrate enhanced SetD7 staining in the 60% Px islets compared with sham controls correlating with β-cell functional compensation. *B*, SetD7 immunofluorescence analysis: Representative pancreatic islet fields from sham control and 60% Px rats were immunolabeled for insulin (*green*), glucagon (*red*), SetD7 (*magenta*), and nuclei (DAPI, *blue*) demonstrating enhanced nuclear SetD7 staining in β-cells of 60% Px rats compared with sham controls under conditions of sustained β-cell compensation. *C*, WB and band quantitation: Lysates from isolated islets from the pancreas of sham & 60% Px rats were analyzed for PPARγ and SetD7. The data are representative of three separate WBs and demonstrate enhanced expression of PPARγ and SetD7 in islets of Px rats compared with sham controls. ∗*p* < 0.002.

We next tested the prediction of parallel changes in islet SetD7 and PPARγ expression *in vivo* using normoglycemic and hyperglycemic partial pancreatectomy (Px) rat models (23, 36). We have previously shown that islet PPARγ expression surges at 5 days postsurgery and is maintained at heightened levels until the second week following a 60%-Px in rats along with preserved normoglycemia as a result of compensatory increases in β-cell mass and function (23). Further, increased PPARγ leads to transcriptionally enhanced *Pdx1* and *GIPR* expression (25, 26). Based on the *in vitro* data in Figure 1, Figure 2, Figure 3 supporting direct transcriptional regulation of *SetD7* by PPARγ, we performed immunostaining analyses of pancreatic sections from sham-operated/Px rats 14 days postsurgery for SetD7 and the islet cell markers insulin and glucagon. We observed markedly increased SetD7 expression and its nuclear enrichment in 60%-Px islets (Fig. 5, *A* and *B*) that was corroborated with islet WB analysis showing increased SetD7 protein (2-fold) and increased PPARγ protein levels (1.9-fold) (Fig. 5*C*).

**Figure 6:**
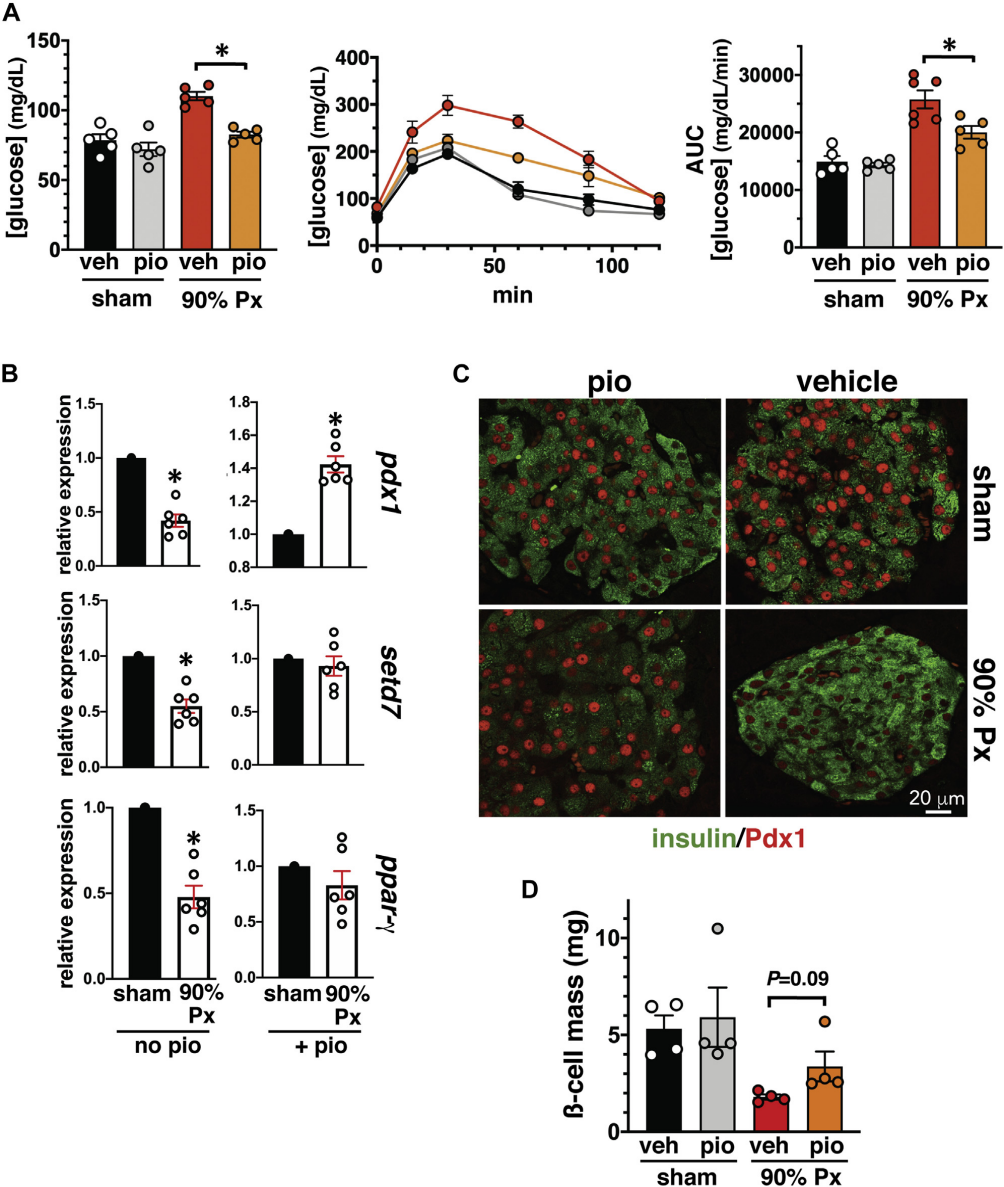
**PPARγ and its targets Pdx1 and SetD7 are downregulated in islets of hyperglycemic 90% Px rats**. Rats undergoing sham and 90% Px were supplemented with or without pioglitazone (2.5 mg/kg in a formulated diet) daily for 3 weeks. *A*, improvement of fed blood glucose levels and glucose tolerance in the 90% Px with pioglitazone compared with 90% Px vehicle group (∗*p* < 0.004) and as shown by IPGTT curves and corresponding AUC plot (∗*p* < 0.02). *B*, RT-PCR gene expression analysis: Total RNA preparations were processed for RT-PCR for the expression of *Pdx1*, *Setd7*, *and PPARγ* using FAM-tagged rat PCR primers. The CT values were normalized with β-actin. Whereas downregulation of *Pdx1*, *Setd7*, *and PPARγ* is observed in islets from 90% Px rats compared with sham controls, pioglitazone supplementation increased Pdx1 mRNA compared with the corresponding sham group and restores the levels of *Setd7* and *PPARγ* to that of the sham group treated with pioglitazone (∗*p* < 0.005). *C*, representative confocal immunofluorescence images of pancreatic islets of sham/Px groups ± pioglitazone; Pdx1 (*red*) and insulin (*green*) immunostaining demonstrates markedly reduced β-cell Pdx-1 in control 90% Px hyperglycemic rats but enhanced nuclear Pdx1 staining with a pioglitazone-supplemented diet. *D*, β-cell mass: Pioglitazone intervention in 90% Px rats shows a trend in partially restoring β-cell mass compared with the control 90% Px group. *p* = 0.09. The data presented represents n = 5 for gene expression, n = 5 for fed glycemia and IPGTT, and n = 3 or 4 for β-cell mass.

In contrast, detrimental effects of hyperglycemia are presumed to be one of the most significant factors driving β-cell dysfunction (1, 2, 3, 4, 5). PPARγ protein expression is markedly downregulated in islets cultured in high glucose media as well as in the islets of diabetic mice (33). We tested *PPARγ* and *SetD7* expression in 90%-Px normal rats that is a well-characterized model of hyperglycemia along with a significant reduction of the key transcription factor Pdx1 (36, 37). We performed sham/90%-Px surgery on 5-week-old male Sprague Dawley rats and intervened with a diet supplemented with normal chow or pioglitazone (2.5 mg/kg daily) for 3 weeks. After 3 weeks, as expected, the 90% Px vehicle control rats maintained modest hyperglycemia and glucose intolerance compared with the sham control and sham pioglitazone groups (Fig. 6*A*). However, the 90% Px surgery on rats treated with pioglitazone exhibited restored normoglycemia and improved glucose tolerance compared with 90% Px control rats (Fig. 6*A*). RT-PCR analysis of isolated islets demonstrated marked reductions in the islet expression of *Pdx1* (0.42-fold), *Setd7* (0.55-fold), and *PPARγ* (0.48-fold) compared with those of the sham controls (Fig. 6*B*, left panel). The 90% Px rats with pioglitazone intervention group exhibited increased islet *Pdx1* (1.42-fold), *SetD7* (0.93-fold), and *PPARγ* (0.82-fold) relative to sham vehicle with pioglitazone treatment (Fig. 6*B*, right panel). Confocal microscopy analysis of 90%-Px islets demonstrated a marked reduction in nuclear Pdx1 immunostaining that was restored after 3 weeks of pioglitazone treatment (Fig. 6*C*). Finally, compared with 90%-Px, there was a trend toward recovery (>40%) of β-cell mass in pioglitazone-treated 90%-Px rats (Fig. 6*D*). These data support the notion that hyperglycemia downregulates *Pdx1*, *PPARγ*, and *Setd7* and the transcriptional activation of *PPARγ* directly upregulates the downstream gene targets *Pdx1* and *Setd7* with partial restoration of functional β-cell mass.

## Discussion

Numerous studies have demonstrated that defects in β-cell compensation are paramount in the transition from a normoglycemic, insulin resistance state to decompensation resulting in frank diabetes (1, 2, 3, 4, 5). As a basis for understanding the molecular regulation of normal and defective β-cell function, many transcription factors (TFs) have been identified that orchestrate various aspects of β-cell development and maintenance of postnatal β-cell mass and function (6, 7, 38, 39, 40). The current results provide *in vitro* and *in vivo* evidence for molecular interactions between the TF/nuclear hormone receptor PPARγ (NR1C3) and the epigenetic factor SetD7 along with Pdx1 as core components of the β-cell adaptive response. The results were obtained in rodent models and rodent-derived β-cell lines. Importantly, a conserved PPRE was also observed in the human *SetD7* promoter sequence. SetD7 is a lysine methyltransferase (KMT) that methylates histone H3 at lysine 4 (H3K4) and several nonhistone TFs that regulate a variety of cellular functions at the transcriptional level (15, 16, 17). Expression is mainly restricted to pancreatic islets (β-cells, α-cells, and some duct cells) in the mature murine and human pancreas (16, 17). Prior studies have identified SetD7-mediated posttranslational methylation of H3K4 on β-cell-enriched genes including *Pdx1*, *Nos2*, Ins1/2, Glut2 and play critical role in glucose sensing, insulin secretion along with modulating ER stress and inflammatory responses (16, 18, 34, 41). Additionally, SetD7 also catalyzes the posttranslational methylation of several key transcription factors (Pdx1, p53, NFκB, TAF10, FXR, androgen and estrogen receptors, FoxO3) that enhance TF stability, function, and DNA interactions (15, 18, 42). Collectively, these findings support an essential regulatory role of SetD7 to maintain the transcriptionally active status of key genes.

We and others have previously shown that *Pdx1* (25), *GIPR* (26), Glut2 (28), *Gck* (29), *and Serca2A* (32) genes are transcriptionally regulated in β-cells by PPARγ binding to promoter specific response elements. Here, we have provided evidence for direct transcriptional regulation of SetD7 by PPARγ in support of our data showing enhanced SetD7 promoter reporter activity and protein expression in the presence of the *PPARγ* transcriptional agonist pioglitazone. In parallel, we observed increased Pdx1 protein expression and promoter reporter activity for the *Pdx1* PPRE that is an established transcriptional target of PPARγ in β-cells (25). A similar increase in SetD7 and Pdx1 levels has been reported in *db/db* mouse β-cells treated with pioglitazone, however, at the time, without a clear mechanistic understanding of the PPARγ-SetD7 interaction (34).

Our findings include the identification of conserved putative PPREs in the mouse, rat, and human *SetD7* promoter sequences. Direct transcriptional regulation of *SetD7* by PPARγ was supported by a nuclear-binding assay (EMSA) of the *SetD7* PPRE sequence in β-cell nuclear extracts, with a band shift that was confirmed to be PPARγ by a super-shift assay. *In situ* occupation of *SetD7* promoter by PPARγ was confirmed by a ChIP assay using PPARγ IP antibody followed by detection of the expected 144 bp PCR band using primer pairs in the flanking regions of the *SetD7* PPRE promoter sequence. Furthermore, a stable constitutive knockdown of PPARγ in INS-1 cells resulted in the downregulation of SetD7 and Pdx1 proteins and corresponding promoter reporter activity coincident with loss of pioglitazone responsiveness.

There is considerable overlap of identified downstream targets of PPARγ and SetD7 actions. PPARγ directly regulates transcriptional activation of *Pdx1* (25), Glut2 (28), *Gck* (29), and alterations of *SetD7* also result in downregulation of the similar targets (17, 18). This led us to study the impact of constitutive KD of *SetD7* in INS-1 cells. Our data demonstrated that effective KD of *SetD7* also resulted in the downregulation of the master transcription factor Pdx1 that coincided with attenuated GSIS similar to previous reports (18). SetD7-mediated posttranslational methylation of Pdx1 is reported to enhance transcriptional activity and its protein stability, hence, SetD7 depletion may also have directly led to the downregulation of Pdx1 (18). Also, *SetD7* KD directly affecting Pdx1 levels reflects that biological pathways have multiple mechanisms to fine-tune gene expression. Interestingly, the measurement of INS-1 cell proliferation by BrdU incorporation in the *SetD7* KD cells demonstrated enhanced proliferation. It appears that SetD7 KD resulting in downregulation of key β-cell maturation and differentiation marker Pdx1 resulted in the loss of functional maturity, but enhanced proliferation. These observations are similar to a recent report suggesting that priming insulin-producing cells to enter the cell cycle promotes a functionally immature phenotype (43). A prodifferentiation role of SetD7 is also demonstrated in analogous tissues such as the muscle, stem cells, and cardiomyocytes (44, 45, 46).

We have extensively characterized the temporal sequences of molecular events and physiological parameters of glucose homeostasis in C57BL/6NTac mice fed a 60% HFD *versus* a control diet for 16 weeks (35). The HFD mice develop progressive obesity, insulin resistance, hyperinsulinemia, and glucose intolerance (35). Next, we evaluated changes in the expression of *SetD7* in the islets of this model of metabolic stress. The HFD mice develop glucose intolerance within a week that coincided with reduction in expression of *SetD7* along with previously reported decreased expression of *MafA*, *Glut2*, *Gck*, *GIPR*, *and PPARγ* in islets (35). However, SetD7 expression is restored to the control levels by fourth week on HFD preceded by a progressive increase in *PPARγ* expression at 2–3 weeks (35). The metabolic adaptive response to HFD included maximal expression of *SetD7* and other key β-cell profunction genes, including *PPARγ*, at 8 weeks. Importantly, *SetD7* downregulation at 12 and 16 weeks of HFD is marked by a decompensatory phase of the β-cell response under persistent metabolic stress. Although these gene expression data are correlative in nature, still support a putative role of *SetD7* in the adaptive β-cell response under metabolic stress. We have also observed nuclear depletion of SetD7 immunoreactivity in the pancreatic sections of both diabetic *db/db* mice and Zucker diabetic fatty rats compared with corresponding controls (data not shown). A 6-week pioglitazone intervention study in *db/db* mice resulted in significant decrease in circulating free fatty acids, triglycerides, and cholesterol; however, *db/db* mice gained weight (34). Further, these mice showed improved glucose tolerance and higher blood insulin levels without changes in insulin sensitivity (34). Pioglitazone in clinical use is thought to enhance peripheral insulin sensitivity as a diabetes therapeutic agent (47, 48); however, these supportive data also demonstrate direct effects of PPARγ agonists in the functional preservation of β-cells (34).

Further *in vivo* evidence for molecular interaction of *PPARγ* and *SetD7* was obtained using a pancreas-specific (PANC) *PPARγ*^−/−^ knockout mouse that we previously characterized and observed to display glucose intolerance, but no change in β-cell mass (25). These mice also exhibited decreased glucose-stimulated insulin secretion along with reduced expression of *Pdx1* and *Glut2* genes (25). We have demonstrated by immunostaining and WB that islets from PANC *PPARγ*^−/−^ mice displayed reduced islet SetD7 immunoreactivity. Also, our group has extensively characterized the adaptive β-cell response that allows preserved normoglycemia in a 60% pancreatectomy (Px) rodent model (19, 20, 21, 22). The adaptive response in this model has three distinct phases: (i) increased β-cell proliferation in the first week post Px in tandem with activation of the IRS2-PI3K-Akt pathway; (ii) second week, activation of β-cell differentiation and functional markers Pdx1, Nkx6.1, GIPR, and pyruvate carboxylase (PC) along with nuclear enrichment of PPARγ; and (iii) attainment of functional maturation (3–4th week) characterized by β-cell hyperfunction associated with enhanced catalytic activity of glucokinase (19, 20, 21, 22). Analysis of SetD7 expression in isolated islets and pancreas staining from 60% Px rats demonstrated an increase in islet/β-cell SetD7 expression coinciding with the enhanced expression of PPARγ, which we have advocated, drives the functional maturation phase of the adaptive β-cell response post 60%-Px. In contrast, glucotoxicity is one of the most significant factors driving β-cell decompensation with the downregulation of function and survival genes (1, 2, 3, 4, 5). We observed the expected decline of *Pdx1*, *PPARγ*, and *SetD7* gene expression in the islets of hyperglycemic 90%-Px rats compared with sham control islets. Also, importantly, a 3-week pioglitazone diet supplementation restored the islet mRNA levels of *Pdx1*, *PPARγ*, and *SetD7* along with markedly enhanced nuclear presence of Pdx1 that was associated with a partial recovery of β-cell mass.

In summary, we have provided evidence for a direct transcriptional regulation of *SetD7* by PPARγ, and this molecular interaction is manifested during a transient phase of adaptive β-cell response following Px as well as during metabolic stress of HFD-induced obesity and insulin resistance. We have also demonstrated that *SetD7* KD in INS-1 cells causes a functional decline but an increase in cellular proliferation. SetD7 is also reported to induce posttranslational methylation of Pdx1, enhancing its protein stability and transcriptional efficacy (18). We propose that the transcriptional upregulation of *Pdx1* and *SetD7* by PPARγ, combined with SetD7-mediated posttranslational methylation of Pdx1, functions as a mechanism that drives β-cell adaptation mechanisms to preserve functional β-cell mass. Furthermore, the transcriptional activity of PPARγ is regulated by tissue-specific factors. PPARγ is abundantly expressed in adipose tissue where it promotes adipogenesis through direct transcriptional regulation of another SET-domain containing gene, *SetD8* (49). Additionally, during mouse pancreas development, RNA-sequence analyses of β-cells have identified enhanced expression of *PPARγ* and *MafA* at postnatal day 60 that is linked with β-cell functional maturation and nutrient sensing (50). We surmise a prominent role for SetD7 in the functional maturation of β-cell during development that remains to be fully resolved. As such, our study identifies SetD7 as a novel β-cell gene regulated by PPARγ and highlights tissue-specific roles for SET-domain containing methyltransferases.

## Experimental procedures

### Cell culture and *in vitro* assays

INS-1 (832/13) cells (gift from Christopher Newgard, Duke University) were cultured in RPMI 1640 (GIBCO) containing 10% FBS, 10 mmol/L HEPES, 2 mmol/l L-glutamine, 1 mmol/l sodium pyruvate, 100 units/ml penicillin, 100 μg/ml streptomycin, 50 μmol/L β-mercaptoethanol, and 8.3 mmol/L glucose. For some experiments, INS-1 cells were grown (72 h) in the presence of 10 μmol/L pioglitazone (Sigma) or DMSO, with daily media replenishment. Beta-TC-6 (ATCC, CRL-11560) cells were cultured in DMEM (ATCC) with 15% heat inactivated FCS and 100 units/ml penicillin, 100 μg/ml streptomycin.

### Electrophoretic mobility shift (EMSA) assay

Nuclear extracts from INS-1 cells were prepared using the NucBuster kit (Novagen). PAGE-purified, infrared-700 (IRD-700) tagged oligos (IDT Inc.) for the mouse SetD7 PPRE (FW 5′-AACCAAGCCAAAAGGTAAACG-3′; REV 5′-CGTTTACCTTTTGGCTTGGTT-3′) were diluted in 1xTE buffer at the concentration of 20 pmol/μl. The diluted oligos were annealed (100 pmol each) by incubating the tubes at 100 °C for 3 min and cooling slowly. The DNA-binding reaction was performed in a 20 μl reaction mixture containing EMSA buffer (100 mM KCl, 20 mM HEPES, 0.2 mM EDTA, 20% glycerol, 0.5 mM DTT, pH 7.5) and 500 ng sonicated salmon sperm DNA, 0.01 U poly (dI-dC), 10 μg nuclear extract, 1:200 diluted annealed probe, and incubated 1 h at room temperature. Overnight cast 6% nondenaturing DNA retardation gels (29:1 acrylamide to bisacrylamide) were prerun 30 min in EMSA running buffer in 0.5 M Tris, then loaded with reaction mixture (18 μl DNA/protein complex mix, 2 μl 6× DNA loading dye), and samples were separated by electrophoresis 2.5 h at 100 V. For the super-shift gel retardation assay, binding reactions were followed by addition of PPARγ-specific antibody (rabbit polyclonal, Thermo Fisher, Cat no. PA3-821A) or rat IgG (nonimmune serum, Sigma) for another 20 min before resolving the DNA/protein complex on a nondenaturing gel. EMSA gels were imaged using an LI-COR imaging system.

### Chromatin immunoprecipitation (ChIP) assay

Chromatin immunoprecipitation assay was performed with mouse-derived βTC6 cells using ChIP-IT kit (Active Motif). Cells were fixed with formaldehyde, and the chromatin sheared by enzymatic digestion according to the instruction manual to DNA fragments that averaged 300–500 bp in length. A mouse monoclonal antibody against PPARγ (E8, mouse monoclonal, ChIP grade, Santa Cruz Biotechnology) was added to aliquots of precleared chromatin and incubated overnight, with parallel samples incubated with the negative-control mouse IgG provided with the kit. Protein G-agarose beads were added, and the mixture was incubated 1.5 h at 4 °C. After reversing the cross-links, DNA was isolated and PCR reactions were performed with primers for the mouse SetD7 promoter region PPRE. Primer sequences were: (−496 to −477), FW5′-CCGCGTGGCTGGCGAACAGT-3′; (−353 to −376), REV5′-TTGACAGTTCCCTGTCCTTTC CCA-3′ (expected PCR product 144 bp). PCR conditions were 1 cycle of 94 °C for 3 min, 39 cycles of 94 °C for 30 s, 60 °C for 1 min, and 72 °C for 1 min. Input DNA (1:50 dilution) was used as a control in the PCR reaction. In addition, immunoprecipitated chromatin against PPARγ and IgG were used to run parallel PCR reactions using primer pairs (FW: 5′-GGTACATGCGCATGCATTTAACCC-3′; REV:5′-GACTCAGGCATCTTTG-3′; expected PCR product 129 bp) covering a non-PPRE region 800 base pair away on the SetD7 promoter sequence to check the fidelity of ChIP-PCR. PCR conditions were 1 cycle of 94 °C for 3 min, 39 cycles of 94 °C for 30 s, 51 °C for 1 min, and 72 °C for 1 min. Input DNA (1:50 dilution) was used as a control in the PCR reaction.

### Generation of stable INS-1 cells with constitutive knockdown of PPARγ and SetD7

We obtained targeted validated four 29mer shRNA carrying plasmids in a retroviral GFP vector (pGFP-V-RS) directed against rat PPARγ (Origene, TG709717) GI738869: 5′-TGTTCGC CAAGGTGCTCCAGAAGATGACA-3′; GI738870: 5′-AGTGACTCCTGCTCAAGTATGGTGTCCATG-3′; GI738871: 5′-CCAGCAT TTCTGCTCCACACTATGAAGAC-3′; GI738872: 5′-CCTTTACCACGGTTGATTTCTCCAGCATT-3′ and a scrambled control shRNA (5′-GCACTACCAGAGCTAACTCAGATAGT ACT-3′). Four shRNA carrying plasmids in a retroviral RFP vector (pRFP-C-RS) directed against rat SetD7 (Origene, TF08040) had following shRNA sequences: FI732161: 5′-GCACCTGGACGATGACGGATTACCACACG-3′; FI732162: 5′-GACCGTTGCCTACGG CTATGACCACAGCC-3′; FI732163:5′GGTG TTCTGCAAGGCACCTATGTGGATGG-3′; FI732164: 5′-CCTTTACTCCAAACTGCGTCT ATGACATG-3′; scrambled control shRNA: 5′-GCACTACCAGAGCTAACTCAGATAGTACT-3′. These plasmids were propagated in *E. coli* and plasmid DNA was isolated using a midi-prep kit (Qiagen). INS-1 cells (60% confluency, 10 cm plates) were transfected with either a single or pooling of four shRNAs (20 μg plasmid DNA along with a scrambled control using Lipofectamine-2000; Invitrogen). After a 24 h posttransfection period, the INS-1 cells underwent puromycin selection (2.5 μg/ml) for 3 weeks. The puromycin-resistant/GFP-positive clones expressing PPARγ shRNAs or scrambled shRNA and puromycin resistant/RFP-positive clones expressing SetD7 shRNAs or scrambled shRNA lifted using cloning cylinders (Sigma). We screened approximately 30 clones for stable KD of PPARγ by WB screening and obtained a single clone yielding a 63% reduction of PPARγ protein compared with the scrambled control. Although, all the shRNA constructs against SetD7 demonstrated reduction in SetD7 expression, however, INS-1 cells expressing shRNA construct FI732164 caused the highest levels of reduction in SetD7 protein (66% compared with scrambled shRNA) and subsequently used in all the experiments. These stable shRNA clones of INS-1 cells were maintained under puromycin selection (1 μg/ml) and used for the subsequent experiments.

### Luciferase reporter gene assay

Sixty percent confluent INS-1 cells in 6-well plates were incubated overnight in antibiotic-free media. INS-1 cells were transfected with 3× PPRE-luciferase reporter vector (Dr Speigelman Lab, Addgene), pTAL-PPRE-Pdx-1 promoter vector (51) (firefly luciferase vector, Dr Roland Stein, Vanderbilt University) or SetD7-PPRE-Luciferase reporter vector (p-Fox-Luc carrying −1 to −1979 SetD7 promoter sequence from the transcription start codon (16), kindly provided by Dr Raghu G. Mirmira, University of Chicago) with Lipofectamine 2000 transfection reagent (Invitrogen). *Renilla* luciferase reporter plasmid (pRL-TK, Promega) was included (1:50) in all transfections as an internal control. Twenty-four hours posttransfection, cells were incubated another 48 h with 10 μmol/L pioglitazone or DMSO. Cells were lysed using passive lysis buffer and cell lysates were used for the luciferase reporter assay performed in a TD 20/20 luminometer (Turners Design) using a dual luciferase assay kit (Promega). Firefly luciferase activity was normalized with *Renilla* luciferase and expressed as relative luciferase activity. Each experimental condition was performed in triplicate, with the results calculated as relative (%) luciferase value of the DMSO-treated respective controls.

### Measurements of protein and mRNA expression

The INS-1 cells and isolated islets were washed with cold 1xPBS thrice and lysed using lysis buffer (50 mmol/L HEPES, 150 mmol/L NaCl, 1% Triton-X 100, 5 mmol/L EDTA, 5 mmol/L EGTA, 20 mmol/L sodium pyrophosphate, 20 mmol/L sodium fluoride, 1 mmol/L activated sodium orthovanadate, 1 mmol/L PMSF, and 1× protease inhibitor cocktail containing 1 μg/ml each leupeptin, aprotinin, and Pepstatin A, pH 7.5). INS-1 cell lysates or islet proteins (25 μg) were separated by SDS-PAGE and electrophoretically transferred onto PVDF membranes (Bio-Rad Laboratories). Membranes were incubated overnight at 4^o^C with one of the following antibodies: PPARγ (1:1000 dilution in 5% PBST-milk, rabbit polyclonal, Thermo Fisher, Cat no. PA3-821A), Pdx1 (1:1000 dilution in 5% PBST-milk, rabbit polyclonal, Millipore, Cat no. 06-1379), SetD7 (1:1000 dilution in 5% PBST-milk, rabbit polyclonal, Cell Signaling, Cat No. 2813), Immunoblots were further processed with washing and 1 h incubations with secondary antibodies and detection by chemiluminescence (Western Thunder, ThermoFisher) and exposure using HyperFilm-ECL (Amersham). Immunoblots were stripped and reprobed to establish equivalent loading using anti-β-actin (Cell Signaling, Cat No. 4967). Protein band intensities were quantitated using NIH-ImageJ software.

Total RNA was isolated from handpicked islets using an RNeasy micro kit (Qiagen) with a single step on-column DNase digestion. The quality and quantity of total islet RNA were analyzed by NanoDrop spectrophotometry (ThermoFisher) and total RNA (1 μg) was reverse transcribed (ImProm-II reverse transcription system, Promega), and the resulting cDNA was subjected to quantitative real-time PCR analysis (QuantStudio3, Applied Biosystems). Fluorescein amidite (FAM) labeled primer probes (Applied Biosystems) were used to amplify: *Pdx1* (*Rn00755591*), *SetD7* (*Rn4331182*), *and PPARγ* (*Rn00440945*). The threshold cycle (C_T_) method was used to determine relative enrichments of respective mRNAs of enlisted genes. All the C_T_ values of sample genes were normalized for total RNA using C_T_ values of *ActinB* (*Rn00667869*) message levels. The gene expression data presented as the average of triplicate gene expression determinations in three independent experiments.

### Measurements of cellular proliferation and glucose-stimulated insulin secretion (GSIS) in INS-1 cells with constitutive KD of SetD7

The INS-1 cells expressing scrambled control and *SetD7* shRNA were cultured in the complete RPMI media on cell culture treated chamber glass slides (BD Falcon) to reach 70% confluency. The cultured cells were exposed to RPMI media containing 10 μM 5-bromo-2-deoxyuridine (BrdU: Thermo Fisher) for 6 h. The cells were washed three times × 2 min in PBS. The cells were fixed with 3.7% formaldehyde for 30 min followed by three times × 2 min wash in PBS. The cells subsequently underwent 10 min incubations with 1 N and 2 N HCL (Sigma) followed by 10 min incubation with phosphate/citric acid buffer (pH 7.4). The cells were washed with 0.1% Triton X-100 in PBS three times × 2 min before blocking in PBS with 5% normal donkey serum. The cells were incubated overnight with mouse anti-BrdU antibody (mouse monoclonal, Cell Signaling) followed by washing and incubation with biotinylated donkey anti-mouse IgG (Jackson ImmunoResearch). Finally, cells were labeled with streptavidin-AlexaFluor 647 (Jackson ImmunoResearch) and DAPI. BrdU incorporation was assessed by examining a minimum of ∼1200 cells/group in several random 20× microscopic fields on a Nikon Ti-E workstation by counting the number of BrdU+ nuclei divided by the total number of nuclei (cells) counted. The proliferation was measured calculating BrdU^+^ divided by the total number of cells.

The INS-1 cells expressing scrambled control and SetD7 shRNA at 80% confluency were cultured in the complete RPMI media on 24-well cell culture plates for 12 h in RPMI 1640 media at 2.8 mM glucose and then equilibrated for 2 h in warm and oxygenated KRBH pH 7.4 (135 mM NaCl, 3.6 mM KCl, 0.5 mM NaH_2_Po_4_, 0.5 mM MgCl_2_.6H_2_O, 1.5 mM CaCl_2_.2H_2_O, 2 mM NaHCo_3_, 10 mM HEPES) 2.8 mM glucose and 0.5% BSA. Insulin secretion was assessed by stimulating the cells at two different glucose concentrations (2.8 and 16.7 mM) for 1 h in warm and oxygenated KRBH/0.5% BSA at 37 °C. Insulin secretion medium was separated from the cells by centrifuging (3000 rpm 5 min at 4 °C) and stored at –20 °C for insulin secretion measurement by insulin ELISA (Thermo Fisher). Cells were washed three times with PBS followed by lysis and collection of cell lysates. The insulin secretion was normalized to total milligrams of protein content in each well.

### Animal studies

The animal studies outlined were performed following approved animal use protocols and guidelines specified by the UVM Institutional Animal Use and Care Committee (IACUC). The high-fat feeding regimen (1–16 weeks) in C57B6-NTac mice, islet isolation, and gene expression analysis methods are described previously (35). The mRNA expression of *SetD7* was performed by RT-PCR and the data presented are relative to age-matched control mice on normal chow. Mice with PPARγ deficiency restricted to the pancreatic epithelium (PANC PPARγ^−/−^) were generated by crossing Pdx1-cre mice (original source, D. Melton, Harvard University) and mice with two floxed PPARγ alleles as previously detailed (25, 52). Male Sprague-Dawley rats (90–120 g) underwent 60% Px and 90% Px using our previously described protocol (19, 36). Control rats (shams) underwent laparotomy and mobilization of the pancreas. Islets were isolated by pancreas duct infiltration with collagenase, Histopaque gradient separation, and handpicking followed by preparation of cell lysates and total RNA isolation (RNeasy micro kit, Qiagen). The sham/90%-Px operated Sprague Dawley rats were diet supplemented with a normal diet or diet with pioglitazone (Takeda Inc, 2.5 mg/kg daily) for 3 weeks. Fed blood glucose concentrations were measured in the tail nick blood (FreeStyle monitor, Abbott Inc.). Intraperitoneal glucose tolerance test (IPGTT; 2 g/kg) was performed in 6 h fasted experimental rat groups (n = 5) by sampling glucose concentrations at 0, 15, 30, 60, 90, and 120 min post glucose administration.

### Pancreas morphometry and immunofluorescence

Pancreata were excised and immersion-fixing overnight in 4.0% paraformaldehyde in 0.1 mM phosphate buffer at 4 °C. After washing in several changes of PBS, tissues were dehydrated and embedded in paraffin. β-cell morphometric data was acquired and analyzed using a Nikon Ti-E widefield fluorescence workstation and NIS Elements software as described previously (22, 35). For each assay, minimum of 3–4 rats per group were measured. For multiple-labeling immunofluorescence studies of SetD7 and Pdx1, sections were stained with guinea pig anti-insulin (Linco/Millipore) to mark β-cells, mouse anti-Pdx1 (Developmental Studies Hybridoma Bank, University of Iowa), and anti-SetD7 (mouse monoclonal, Abcam, Cat No. Ab14820). Secondary antibodies consisted of multiple-labeling grade anti-species-specific IgG conjugated to CY2, CY3, or AlexaFluor 647, respectively (Jackson ImmunoResearch or Molecular Probes/Invitrogen). Some sections were imaged confocally using an LSM 510 META (UVM Microscopy Imaging Center).

### Statistical analysis

Data are presented as mean ± SEM or SD, as indicated. Each data point from the animal studies represents an individual animal. Statistical significance was determined by the unpaired Student's *t* test or one-way ANOVA (GraphPad Prism). For all data, significance was determined as *p* < 0.05, and a trend was considered as *p* = 0.06–0.09

## Conflict of interest

The authors declare that they have no conflicts of interest with the contents of this article.
